# Associations Between Human Exposure to Substituted *p*-Phenylenediamine-Derived Quinones and Colorectal Cancer Risk

**DOI:** 10.3390/toxics14080705

**Published:** 2026-08-10

**Authors:** Zhenling Fu, Zefu Hu, Hangbiao Jin, Sihui Yin

**Affiliations:** 1 Department of Pharmacy, Quzhou People’s Hospital: The Quzhou Affiliated Hospital, Wenzhou Medical University, Quzhou 324000, China; 276784489@wmu.edu.cn (Z.F.); huzefu@wmu.edu.cn (Z.H.); 2 State Key Laboratory of Green Chemical Synthesis and Conversion, Zhejiang Key Laboratory of Clean Energy Conversion and Utilization, Science and Education Integration College of Energy and Carbon Neutralization, Zhejiang University of Technology, Hangzhou 310014, China; hangbiao@zjut.edu.cn

**Keywords:** PPD-Qs, 6PPD-Q, urinary level, colorectal cancer risk, mixed exposure

## Abstract

Substituted *p*-phenylenediamine-derived quinones (PPD-Qs) have become ubiquitous contaminants in various environmental matrices. However, the understanding of the potential health risks posed by human exposure to these emerging compounds remains limited. In this case–control study, we investigated the association between urinary concentrations of six PPD-Q congeners and colorectal cancer (CRC) risk. Detection frequencies of all target PPD-Qs in human urine ranged from 73.7% to 88.2% in the control group and from 70.2% to 89.6% in the case group. 6PPD-Q (2-((4-methylpentan-2-yl)amino)-5-(phenylamino)cyclohexa-2,5-diene-1,4-dione; mean 1.37 μg/g creatinine), 77PD-Q (2,5-bis((5-methylhexan-2-yl)amino)cyclohexa-2,5-diene-1,4-dione; 0.68 μg/g creatinine), and IPPD-Q (2-(isopropylamino)-5-(phenylamino)cyclohexa-2,5-diene-1,4-dione; 0.70 μg/g creatinine) were the predominant PPD-Qs in urine from healthy controls. In CRC cases, 6PPD-Q had the highest mean urinary level (mean 1.75 μg/g creatinine), followed by DPPD-Q (2,5-bis(phenylamino)cyclohexa-2,5-diene-1,4-dione; 0.83 μg/g creatinine) and IPPD-Q (0.73 μg/g creatinine). After multivariable adjustment, higher urinary levels of 6PPD-Q (OR for Q4 vs. Q1 = 1.79, 95% CI: 1.26–2.32), CPPD-Q (2-(cyclohexylamino)-5-(phenylamino)cyclohexa-2,5-diene-1,4-dione; OR = 1.60, 95% CI: 1.26–1.94), and DPPD-Q (OR = 1.47, 95% CI: 1.25–1.68) were significantly associated with increased CRC odds, with nonlinear exposure–response patterns observed. Mixture analysis suggested a positive joint association between urinary PPD-Q concentrations and the odds of CRC, with 6PPD-Q and DPPD-Q contributing most strongly to the estimated statistical association. These findings highlight the need for further prospective studies and potential regulatory considerations to mitigate public health impacts from these contaminants.

## 1. Introduction

The significant expansion in industrial tire production, driven by the rising global demand for private vehicles (especially electric cars) [1,2], relies heavily on substituted *p*-phenylenediamines (PPDs) [3,4]. These chemicals are essential antioxidants to enhance tire durability by preventing cracking and thermo-oxidative degradation [5,6,7]. Beyond tires, PPDs serve as stabilizers in a wide array of rubber products, such as protective clothing, wires, athletic equipment, industrial hoses, and electrical insulation [3,8,9]. Some PPDs were designated as high-volume industrial chemicals in 2015 [10], reflecting their substantial global use and commercial significance. This large-scale utilization inevitably leads to the pervasive environmental release of PPDs [11,12]. Once in the environment, PPDs undergo oxidative and photochemical transformation, generating several transformation products, most notably PPD-quinones (PPD-Qs) [13]. Monitoring data have confirmed the widespread presence of these PPD-Q contaminants, including 6PPD-Q (2-((4-methylpentan-2-yl)amino)-5-(phenylamino)cyclohexa-2,5-diene-1,4-dione), CPPD-Q (2-(cyclohexylamino)-5-(phenylamino)cyclohexa-2,5-diene-1,4-dione), and DPPD-Q (2,5-bis(phenylamino)cyclohexa-2,5-diene-1,4-dione), across diverse environmental matrices [12,13,14].

Environment presence of various PPD-Qs facilitates multiple routes of human exposure, including inhalation, dietary intake, and dermal absorption [13,15]. Monitoring studies have consistently detected these compounds in human biological samples (serum, urine, milk, and cerebrospinal fluid) [16,17,18]. Supporting evidence from toxicological research indicated that PPD-Q exposure induced dose-dependent adverse effects, such as hepatic toxicity, intestinal damage, and impaired reproductive function [12,19,20,21]. These findings suggest that PPD-Qs may harm environmental ecosystems and simultaneously present growing risks to public health [22,23]. These concerning findings highlight the necessity for additional studies to determine the health hazards that PPD-Qs may present to humans.

Colorectal cancer (CRC) ranks as a leading digestive tract malignancy, and a significant contributor to the global cancer incidence and mortality, ranking third in incidence and second in cancer-related mortality globally [24,25]. This disease constitutes a serious public health challenge globally [26]. In China, CRC prevalence has shown a consistent annual increase [27,28]. Furthermore, the total medical and hospitalization expenses for CRC patients in China are the second highest among all malignant tumors, surpassed only by lung cancer [29,30]. Identifying the risk factor is crucial for reducing CRC incidence and mortality. Although the precise causes of CRC are not fully known, factors such as family history, physical inactivity, poor diet, obesity, prolonged sedentary behavior, smoking, and alcohol consumption have been linked to its development [31,32]. Despite that, the potential role of emerging environmental contaminant exposure in colorectal cancer development remains poorly understood [33,34].

Toxicological evidence had suggested that 6PPD-Q might negatively impact human gut health. For example, research data on *Caenorhabditis elegans* demonstrated that long-term low-level exposure to 0.1–10 μg/L 6PPD-Q increased the reactive oxygen species (ROS) concentrations, heightened autofluorescence, and increased intestinal permeability [35]. Furthermore, exposure to 1 μg/L and 10 μg/L 6PPD-Q significantly reduced the *acs-22* gene expression level, which could greatly regulate the permeability of the intestinal [35]. These studies indicate that 6PPD-Q may have the potential to promote intestinal oxidative stress and compromise barrier integrity. Although these animal studies imply a potential biological mechanism linking PPD-Q exposure to the CRC risk, epidemiological evidence from human studies remains limited.

This case–control study addresses this critical research gap by characterizing the relationship between human urinary PPD-Q concentrations and CRC risk using an integrated analytical framework. The study cohort comprised 283 newly diagnosed CRC cases and 515 healthy controls. Initial analysis employed logistic regression to evaluate associations between human urinary levels of PPD-Qs and CRC risk. Subsequently, restricted cubic spline (RCS) regression was performed to assess non-linear exposure–response relationships. To account for the complex interactions among multiple PPD-Qs, Bayesian kernel machine regression (BKMR) modeled the mixture effects on CRC risk. By integrating these complementary approaches, the present study aimed to characterize the associations of individual and combined urinary PPD-Q concentrations with the odds of CRC.

## 2. Materials and Methods

### 2.1. Study Population Enrollment

In the present study, enrolling subjects was carried out in 2024 at Quzhou People’s Hospital. The current study comprised 283 individuals newly diagnosed with colorectal cancer as the case group and 515 healthy participants as controls. Eligible participants were required to meet the following criteria: (1) having lived in Quzhou City for at least three years; (2) being able to finish questionnaires and interviews without assistance; and (3) falling within the age range of 40 to 70 years. All colorectal cancer cases were confirmed through colonoscopy and histopathological examination of tissue samples at the same hospital. Controls were recruited from health examination attendees at the hospital during the same study period. Controls were required to have resided in Quzhou City for at least three years. Individuals were eligible as controls if they were 40–70 years of age, had no previous diagnosis of malignant disease or chronic gastrointestinal disease, and had not used gastrointestinal medications during the month preceding enrollment. Control subjects were excluded if they had a history of colorectal cancer, colorectal adenoma, inflammatory bowel disease, or other gastrointestinal malignancies. Written consent was obtained from every participant. The research protocol (Approval No., 2024-172) received approval from Quzhou People’s Hospital.

Trained nurses conducted face-to-face interviews to gather demographic data of all participants. Participants in the case group had not undergone any medical treatments before urine sample collection following their colorectal cancer diagnosis. Control group subjects had no prior diagnoses of chronic gastrointestinal disorders or malignancies, and had not taken any gastrointestinal-related medications in the month preceding the study. Morning fasting urine samples (4–8 mL) were obtained from participants. The collected human urine was promptly divided into aliquots and stored at −80 °C for future testing. To ensure consistency, human urine samples from the case group were collected within three days post-diagnosis, prior to medical interventions or dietary modifications. To assess potential contamination, ultrapure water blanks (*n* = 7) were processed alongside the real human urine samples, undergoing the same aliquoting and freezing procedures.

### 2.2. Analysis of PPD-Qs in Human Urine

The current study focused on analyzing six PPD-Qs in obtained urine specimens, including IPPD-Q (2-(isopropylamino)-5-(phenylamino)cyclohexa-2,5-diene-1,4-dione), DPPD-Q, CPPD-Q, 6PPD-Q, 77PD-Q (2,5-bis((5-methylhexan-2-yl)amino)cyclohexa-2,5-diene-1,4-dione), and DTPD-Q (2,5-bis(o-tolylamino)cyclohexa-2,5-diene-1,4-dione). Their chemical structures are detailed in Table S1 of the Supporting Information (SI). An optimized liquid–liquid extraction protocol was developed to extract PPD-Qs in human urine based on previous methodologies with modifications [36]. For each 2.0 mL human urine aliquot, 6PPD-Q-*d*_5_ (5.0 ng) and benzophenone-*d*_10_ (5.0 ng) were added as internal standards prior to extraction. The extraction procedure involved mixing methyl tert-butyl ether (MTBE; 5 mL) with the urine sample, followed by vigorous vortex mixing for 5 min. After phase separation by centrifugation at 4000× *g* (10 min and 4 °C), the organic supernatant was collected. A second extraction step was performed with an additional 5 mL of fresh MTBE. The combined organic extracts were then concentrated to complete dryness under a controlled nitrogen stream maintained at 20 °C. The final dried residues were finally reconstituted in HPLC-grade methanol (50 μL) and transferred to autosampler vials for subsequent analysis.

This study performed quantitative analysis of PPD-Qs using an UPLC-MS/MS system (ACQUITY UPLC coupled to Xevo TQ-S triple-quadrupole mass spectrometer; Waters Corporation, Milford, MA, USA), and chromatographic separation was achieved on a Hypersil BDS C18 column (50 mm × 2.1 mm i.d., 2.5 μm particle size; Thermo Fisher Scientific, Waltham, MA, USA) maintained at 35 °C. The mobile phase was (A) 0.2% formic acid and 3.5 mM ammonium acetate in ultrapure water and (B) HPLC-grade methanol, delivered at 0.25 mL/min. We optimized the gradient elution program with multiple steps as follows: initial condition of 10% B (0.0–0.50 min), followed by a gradual ramp to 90% B by 10 min (held until 11 min), then rapid return to initial conditions within 0.1 min, with a 3 min re-equilibration period. The mass spectrometer operated in positive electrospray ionization mode with multiple reaction monitoring (MRM), with two specific transitions monitored for each analyte (i.e., quantifier and qualifier ions). Optimal instrument parameters including cone voltages, collision energies, and dwell times for each transition were systematically optimized and are documented in SI, Table S2.

### 2.3. QA/QC

A comprehensive quality assurance program was implemented throughout the analytical process. Potential background PPD-Q contamination was monitored through the analysis of three types of controls, including solvent blanks (pure methanol), procedural blanks (ultrapure water processed identically to samples), and equipment blanks (clean glassware extracts). We injected these blank samples after every ten urine samples. All laboratory reagents and materials were pre-screened to confirm the absence of detectable PPD-Q contamination prior to use.

Quantification was performed for target PPD-Qs using internal standard calibration with matrix-matched standards prepared in PPD-Q-free human urine at six concentration levels ranging from 0.50 to 50 ng/mL. Each calibration curve demonstrated excellent linearity with R^2^ exceeding 0.995. The limits of detection (LODs), defined at a signal-to-noise ratio of three, were used to evaluate method sensitivity [37], which ranged from 0.016 to 0.088 ng/mL across the target analytes. To validate method accuracy, spike-recovery experiments were carried out at three levels (i.e., low, medium, and high) in human urine matrix, yielding recoveries between 81% and 108% for all PPD-Q homologues. Method precision was assessed by analyzing spiked urine samples (five replicates) at 0.50 ng/mL or 5.0 ng/mL, showing intra-day relative standard deviations below 10% and inter-day variability (evaluated over one week) under 15%. Complete method validation data for target PPD-Qs are presented in SI, Tables S3 and S4.

### 2.4. Statistical Analysis

Baseline differences in demographic characteristics, exposure profiles, and lifestyle factors between the two cohorts were assessed using χ^2^ tests for categorical variables (such as gender, occupational status, and smoking habit) and Mann–Whitney *U* tests for continuous parameters (such as age and body mass index). To address the potential bias from left-censored data, urinary PPD-Q levels below the LODs were replaced with LODs/√2. Target analytes demonstrating detection frequencies above 70% were stratified into quartiles (Q1–Q4) using the concentration in the control group. Inter-correlations among individual PPD-Q congener concentrations in human urine were evaluated using Spearman’s coefficients.

Based on the calculated odds ratios (ORs) with corresponding 95% confidence intervals (CI), relationships between human urine PPD-Q levels and colorectal cancer risk were quantified using unconditional logistic regression. To manage right-skewed exposure distributions, PPD-Q concentrations underwent logarithmic transformation prior to analysis as quartile-based categorical variables, using the lowest quartile (Q1) as the reference group to examine incremental risk patterns. Both unadjusted models and multivariable models were constructed. The multivariable models were adjusted for identified covariates (gender, age, BMI, education level, occupation, marital status, fast-food consumption, spicy food consumption, residence, smoking habit, drinking habit, and family history of colorectal cancer), as shown in Table 1. The covariates were chosen *a priori* based on established evidence [38,39]. Dose–response trends were further investigated by assigning the median exposure value within each PPD-Q quartile and incorporating this as a continuous variable in trend tests.

To capture potential non-linear exposure–response dynamics beyond categorical quartiles, restricted cubic spline (RCS) models with three knots (i.e., 5th, 50th, and 95th) were applied. RCS analysis facilitated the graphical representation of continuous exposure–response relationships via smoothed curves, offering deeper insights into the dose–response dynamics of PPD-Q exposure. Furthermore, to evaluate the combined effects of PPD-Q exposure, Bayesian kernel machine regression (BKMR) was employed. This method utilizes kernel functions to model complex multi-pollutant interactions. Prior to BKMR analysis, log-transformed urinary PPD-Q concentrations were *z*-score standardized. To estimate the cumulative effect of the PPD-Q mixture, we compared the predicted CRC risk when all congeners were set to their 75th percentiles simultaneously with the risk at their median baseline concentrations. PPD-Q-specific contributions were isolated by incrementing individual congeners (from 25th to 75th), while holding co-exposures fixed at their median values. Model stability was confirmed over 10,000 Markov Chain Monte Carlo iterations. *R* software (version 4.0.1) was used for statistical analyses, mainly utilizing the “*stats*”, “*rms*”, “*bkmr*”, and “*tidyverse*” packages, with statistical significance defined by a two-tailed *p* level of 0.05.

## 3. Results

### 3.1. Demographic and Lifestyle Characteristics

As summarized in Table 1, the two groups showed broadly similar distributions of gender, age, occupational status, marital status, residence, and spicy food consumption. Case populations had a mean age of 46 ± 14 years, while controls had a mean age of 48 ± 17 years, and the proportions of males were similar between the two groups. Compared with healthy controls, CRC cases had higher proportions of frequent fast-food consumption (34.6% vs. 21.7%; *p* = 0.03), smoking history or current smoking (36.7% vs. 11.1%; *p* < 0.01), drinking history or current drinking (35.7% vs. 13.2%; *p* < 0.01), and family history of colorectal cancer (12.0% vs. 5.6%; *p* = 0.02). In addition, a slightly higher proportion of cases had college-level education compared with controls (27.9% vs. 20.6%; *p* = 0.03).

### 3.2. PPD-Qs in Human Urine

**Figure 1:**
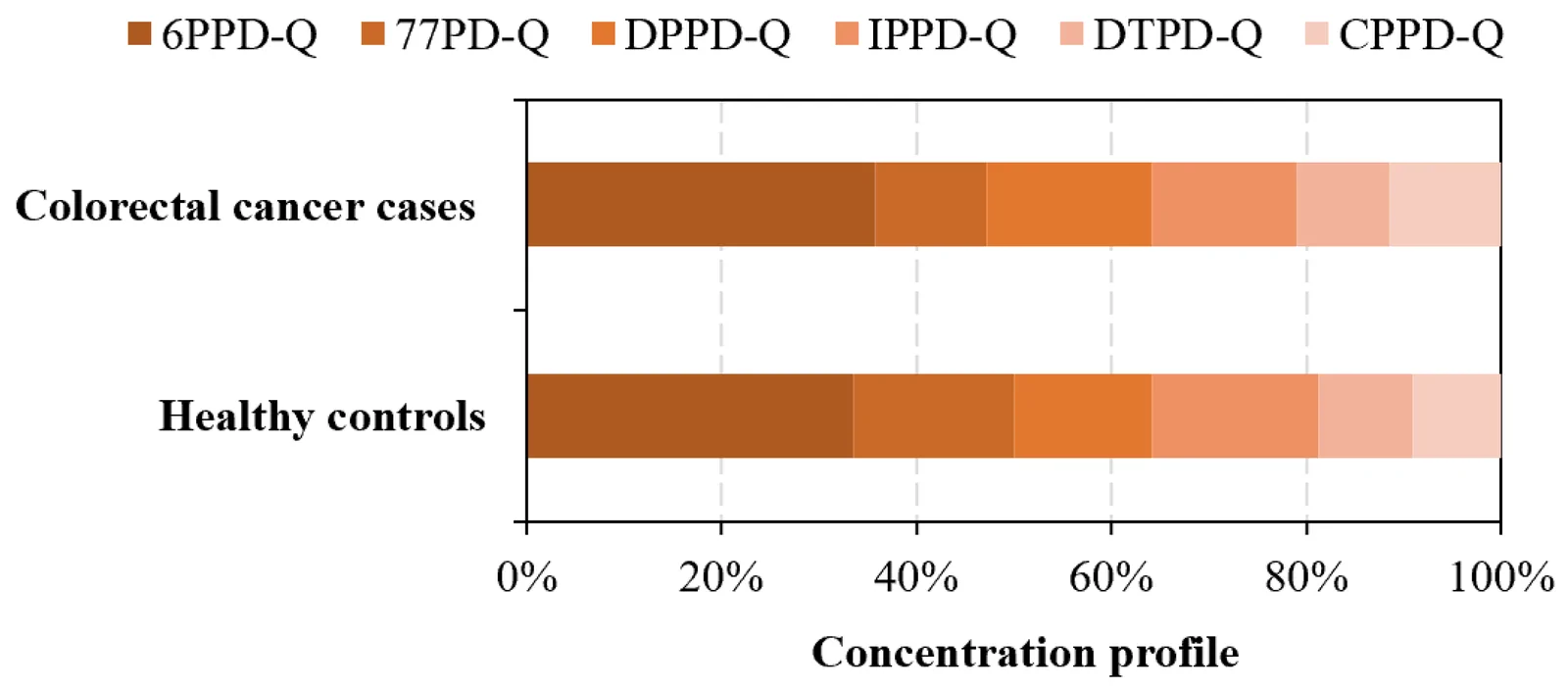
Distribution patterns of PPD-Q concentrations in urine from colorectal cancer cases and control subjects. Note that 6PPD-Q, 2-((4-methylpentan-2-yl)amino)-5-(phenylamino)cyclohexa-2,5-diene-1,4-dione; 77PD-Q, 2,5-bis((5-methylhexan-2-yl)amino)cyclohexa-2,5-diene-1,4-dione; DPPD-Q, 2,5-bis(phenylamino)cyclohexa-2,5-diene-1,4-dione; IPPD-Q, 2-(isopropylamino)-5-(phenylamino)cyclohexa-2,5-diene-1,4-dione; DTPD-Q, 2,5-bis(o-tolylamino)cyclohexa-2,5-diene-1,4-dione; CPPD-Q, 2-(cyclohexylamino)-5-(phenylamino)cyclohexa-2,5-diene-1,4-dione.

Table 2 summarizes the urinary concentrations of six PPD-Q congeners in CRC cases and healthy controls. Overall, all target PPD-Qs were frequently detected in both groups, with detection frequencies of 73.7–88.2% in controls and 70.2–89.6% in cases. Among healthy controls, IPPD-Q showed the highest detection frequency (88.2%), followed by CPPD-Q (82.9%) and 6PPD-Q (79.3%). 6PPD-Q, IPPD-Q, and 77PD-Q were the frequently detected PPD-Qs in urine from cases. The mean concentration in healthy control urine was 1.37 μg/g creatinine (range < LOD–8.30 μg/g creatinine) for 6PPD-Q, 0.70 μg/g creatinine (<LOD–4.06 μg/g creatinine) for IPPD-Q, and 0.68 μg/g creatinine (<LOD–2.68 μg/g creatinine) for 77PD-Q. 6PPD-Q accounted for 33% (mean) of total PPD-Qs in healthy control urine (Figure 1). In CRC cases, 6PPD-Q also had the highest mean urinary level (mean 1.75 μg/g creatinine, range < LOD–13.3 μg/g creatinine). The mean urinary concentrations of DPPD-Q and IPPD-Q were 0.83 μg/g creatinine (range < LOD–3.58 μg/g creatinine) and 0.73 μg/g creatinine (<LOD–7.16 μg/g creatinine), respectively in CRC cases. 6PPD-Q contributed 36% (mean) of total PPD-Qs in CRC case urine.

### 3.3. Associations of Urinary PPD-Q Concentrations with CRC Odds

In both crude and adjusted models, higher urinary concentrations of 6PPD-Q were positively associated with CRC risk (Table 3). Compared with participants in the lowest quartile (Q1), those in the highest quartile (Q4) had a significantly increased CRC risk in the adjusted model (OR = 1.79, 95% CI: 1.26–2.32), with the *p* value for trend < 0.01. For CPPD-Q, the association became more apparent at the highest urinary level, and participants in Q4 showed a significantly elevated CRC risk compared with those in Q1 (adjusted OR = 1.60, 95% CI: 1.26–1.94; *p* for trend = 0.01). A similar pattern was observed for DPPD-Q, for which the adjusted ORs were 1.36 (95% CI: 0.85–2.34) in Q2, 1.22 (95% CI: 1.01–1.43) in Q3, and 1.47 (95% CI: 1.25–1.68) in Q4, accompanied by a significantly positive trend across quartiles (*p* for trend = 0.03).

**Figure 2:**
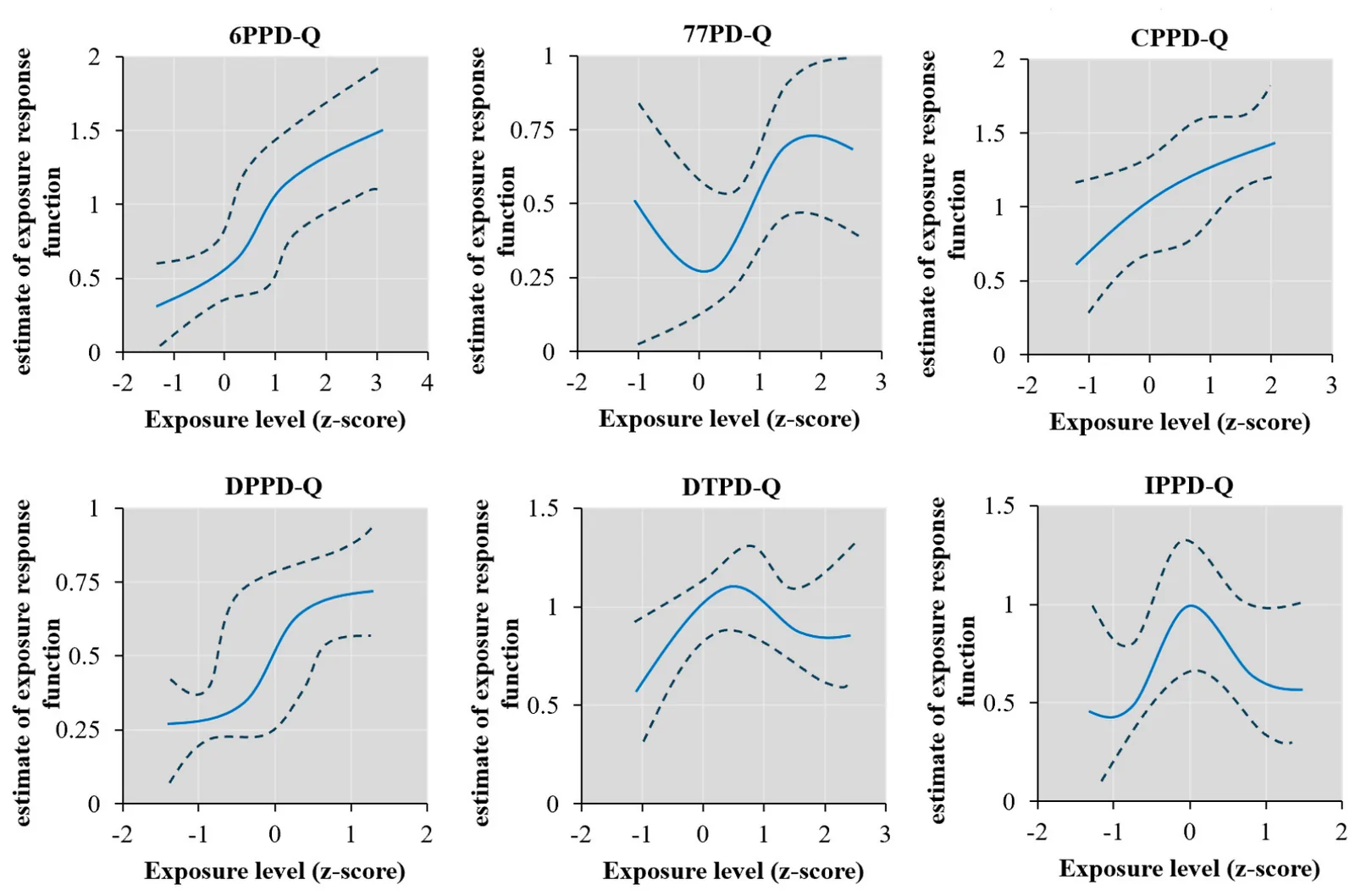
Univariate exposure–response functions with 95% confidence intervals (dashed line region) for individual PPD-Qs. The curves represent adjusted odds ratios (ORs) with 95% confidence intervals (shaded areas) estimated using restricted cubic spline models. Models were adjusted for age, sex, BMI, education level, occupation, marital status, residence, smoking, drinking, fast-food consumption, spicy-food consumption, and family history of colorectal cancer.

**Figure 3:**
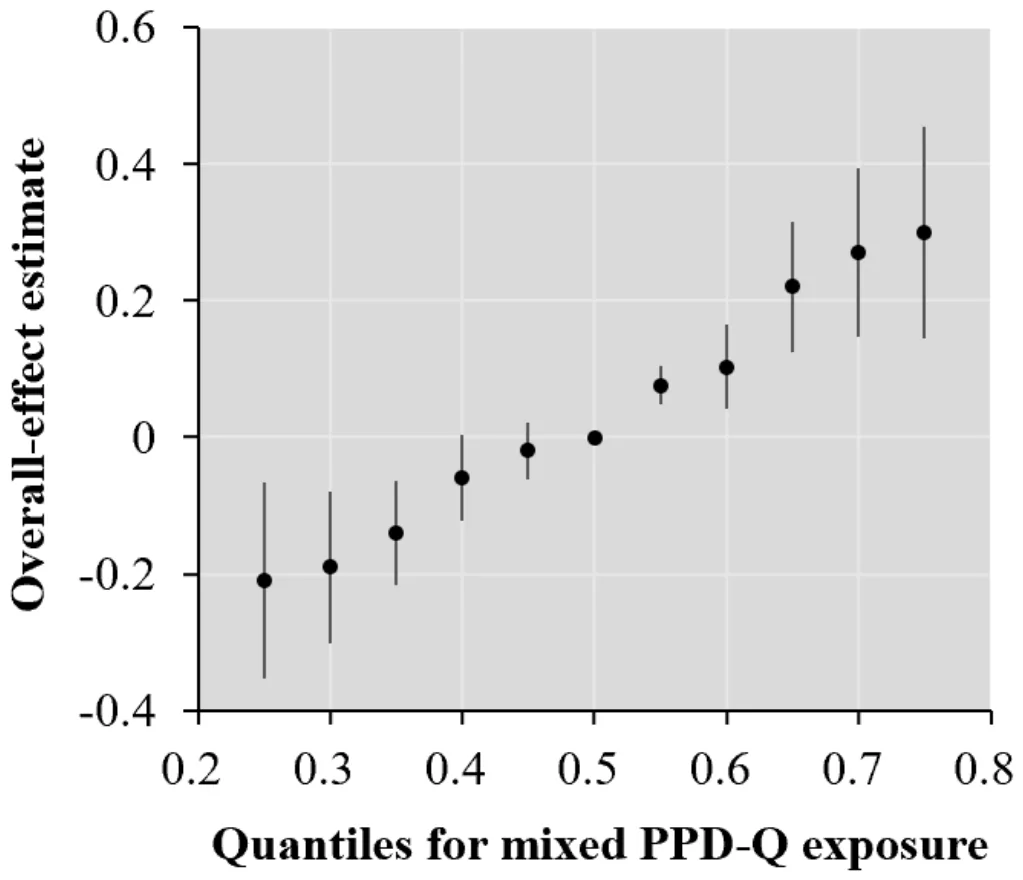
Overall impact of exposure to mixed PPD-Qs on colorectal cancer with 95% confidence intervals for the colorectal cancer case and healthy control groups, estimated using BKMR. Models were adjusted for age, sex, BMI, education level, occupation, marital status, residence, smoking, drinking, fast-food consumption, spicy-food consumption, and family history of colorectal cancer.

The exposure–response curve for 6PPD-Q showed a clearly upward trend across the exposure range, indicating that higher urinary 6PPD-Q levels were associated with the increased estimated CRC risk (Figure 2). A similar positive pattern was observed for CPPD-Q, with the estimated odds of CRC increased across the range of urinary concentrations. DPPD-Q also exhibited an increasing trend, particularly from low to moderate exposure levels, followed by a tendency to plateau at higher levels. In contrast, 77PD-Q, DTPD-Q, and IPPD-Q concentration levels displayed non-linear patterns or non-monotonic relationships with the CRC risk. In addition, Figure 3 shows the overall-effect estimates of mixed urinary PPD-Q exposure, suggesting that elevated joint exposure to PPD-Q congeners may be associated with the increased colorectal cancer risk.

**Figure 4:**
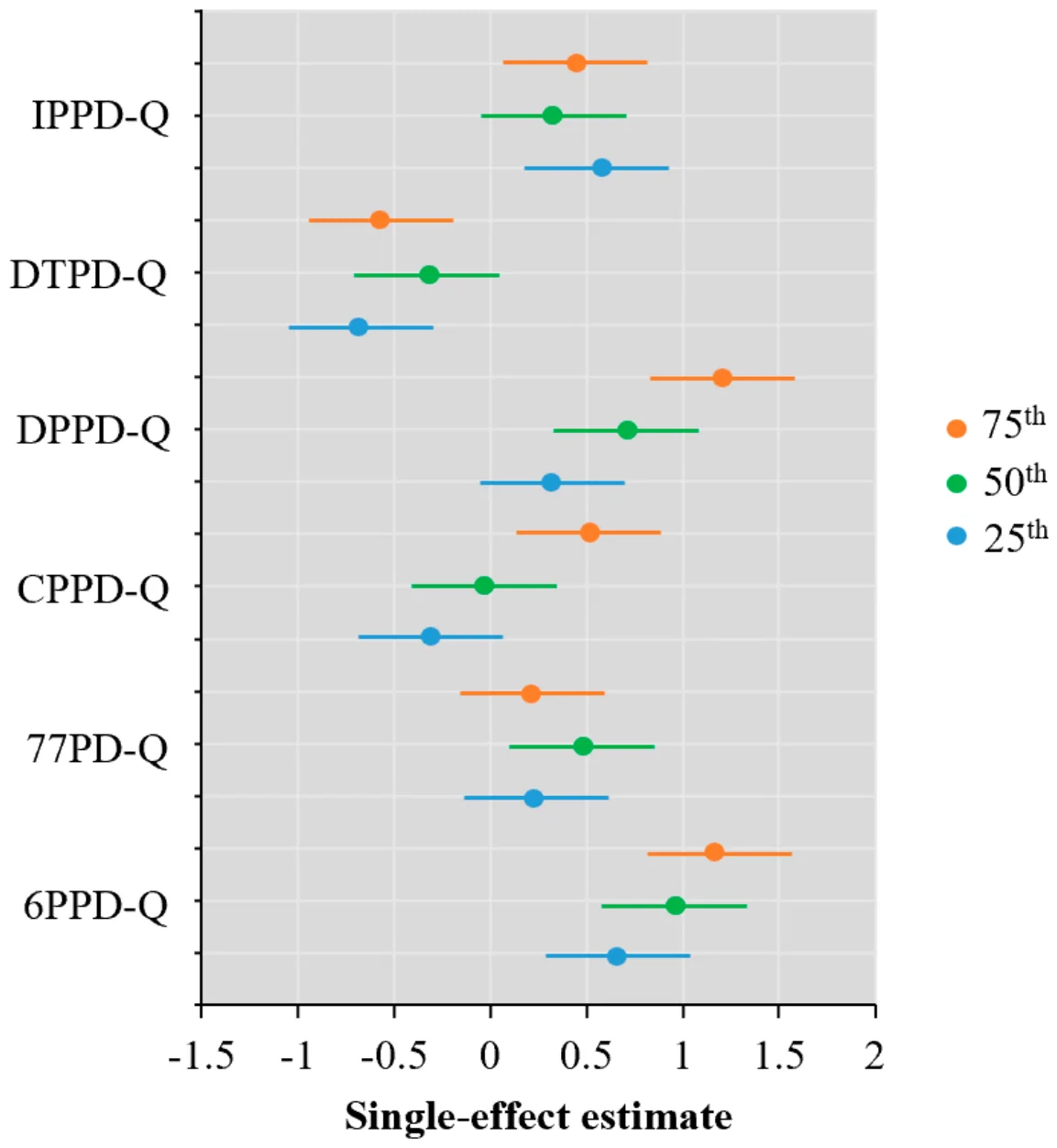
Single-pollutant associations with colorectal cancer for the case and control groups. The plot shows the mixture risk of colorectal cancer examined within each PPD-Q and with all PPD-Qs fixed at 25th, 50th, and 75th percentiles. Positive values indicate increased estimated colorectal cancer odds, whereas negative values indicate decreased estimated odds. All models were adjusted for age, sex, BMI, education level, occupation, marital status, residence, smoking, drinking, fast-food consumption, spicy-food consumption, and family history of colorectal cancer.

Figure 4 presents the single-pollutant effect of individual PPD-Q congeners on colorectal cancer risk. Overall, 6PPD-Q and DPPD-Q showed consistently positive effect estimates across different mixture-exposure levels, with stronger estimates when co-exposures were fixed at the 75th percentile, suggesting that these two congeners contribute most strongly to the estimated positive mixture association. CPPD-Q showed a weaker or near-null effect at lower co-exposure levels but shifted toward a positive association when the other PPD-Qs were held at higher percentiles. In contrast, DTPD-Q generally exhibited negative effect estimates, indicating a different contribution pattern within the mixture. The estimates for 77PD-Q and IPPD-Q were relatively moderate and varied across co-exposure percentiles.

## 4. Discussion

In participant urine samples, multiple PPD-Qs were widely detected, with 6PPD-Q, IPPD-Q, and 77PD-Q as the most abundant PPD-Q congeners. Elevated levels of certain PPD-Qs, including 6PPD-Q, CPDD-Q, and DPPD-Q, in participant urine were associated with higher odds of colorectal cancer. BKMR analysis further revealed a significant positive association between overall exposure to the PPD-Q mixture and CRC risk. Collectively, these results represent the evidence of an association between human PPD-Q exposure and CRC risk.

Among the PPD-Qs detected in human urine, 6PPD-Q was the most prevalent, and other target compounds were also frequently present. Concentrations of PPD-Qs in human urine had been reported in only a small number of previous studies. The reported median urinary 6PPD-Q levels from adults, children, and pregnant women in South China were 0.51 μg/g creatinine (0.078–2.87 μg/g creatinine), 0.093 μg/g creatinine (<LOD–0.64 μg/g creatinine), and 2.76 μg/g creatinine (0.38–9.17 μg/g creatinine), respectively [36]. These levels are comparable to those observed in human urine from this study. The predominant PPD-Qs in human urine from Wuhan, China were DPPD-Q (mean ± SD, 7.2 ± 7.6 ng/mL), CPPD-Q (5.4 ± 5.9 ng/mL), and IPPD-Q (2.2 ± 1.8 ng/mL) [18]. Regional variations in industrial emissions, traffic density, and tire wear particle deposition may result in distinct environmental occurrence patterns of PPD-Q congeners, which in turn affect the urinary PPD-Q concentrations observed in different populations. Meanwhile, differences in demographic characteristics (e.g., age) and lifestyle factors (e.g., dietary habits and smoking) could influence both the extent of PPD-Q exposure and the metabolic clearance of PPD-Qs, thereby contributing to the heterogeneity between study cohorts. Furthermore, methodological discrepancies may also lead to divergent detection frequencies and quantified levels of individual PPD-Q homologues across different studies.

Positive associations between urinary levels of 6PPD-Q and its analogs (e.g., CPPD-Q and DPPD-Q) and CRC risk revealed in the present study are novel. Several biological processes may provide plausible hypotheses for the observed associations, although none can be regarded as an established pathway based on the current evidence. Notably, most available evidence regarding PPD-Q toxicity is derived from in-vitro experiments, non-human models, transcriptomic analyses, or computational predictions, and its relevance to environmentally exposed human populations remain uncertain. One plausible mechanism is the induction of reactive oxygen species (ROS) production, which may subsequently result in oxidative damage. The quinone moiety in the PPD-Q molecular may participate in futile redox cycling, leading to the production of superoxide anions and other ROS [40,41]. This can overwhelm cellular antioxidant defenses, resulting in oxidative stress. Crucially, oxidative stress is a well-established driver of carcinogenesis [42,43]. Persistent oxidative DNA damage may initiate genomic instability, a hallmark of cancer, and may promote the transformation of normal epithelial cells in the colon [44]. In addition, previous research had indicated that PPD-Q exposure could inhibit phosphoinositide 3-kinase (PI3K)/AKT signaling in-vivo and in-vitro [45,46]. Cellular proliferation, growth, and survival are critically regulated by the PI3K/AKT signaling pathway, and its dysregulation is frequently implicated in colorectal carcinogenesis [47,48]. Furthermore, network toxicology analyses had identified the PI3K-AKT pathway as a target of 6PPD-Q in cancer models [49,50]. Chronic suppression of this pro-survival pathway could paradoxically create a selective pressure for cells that acquire mutations to bypass this inhibition, promoting the outgrowth of malignant clones [51,52]. Network toxicology and transcriptomic studies had also identified the JAK-STAT and FoxO signaling pathways as potential targets of 6PPD-Q [53,54]. The JAK-STAT pathway is crucial for mediating inflammatory responses and cell proliferation, both of which are intimately linked to CRC development and progression [55]. Dysregulation of these pathways by PPD-Q may create a pro-inflammatory and proliferative microenvironment conducive to tumorigenesis. Finally, oral exposure to 6PPD-Q had been shown to induce gut microbial dysbiosis in murine models, altering the abundance of specific bacterial genera [40,46]. Given the established role of intestinal microbiome in CRC development (through the production of genotoxins, modulation of the immune system, and alteration of host metabolism) [56,57], this may represent a compelling indirect mechanism. The disruption of gut homeostasis by PPD-Qs may promote an environment favoring the growth of pathogenic bacteria and pro-inflammatory states, thereby increasing CRC risk. The biological mechanisms discussed above provide potential plausibility for the observed associations, but should not be interpreted as evidence that these pathways mediate the associations identified in the present study. Most of the available mechanistic evidence is derived from experimental models, and its relevance to urinary PPD-Q concentrations and CRC in humans remains uncertain. These findings underscore the urgent need for further toxicological and mechanistic studies to delineate the precise pathways.

Several limitations of this study should be acknowledged when interpreting the findings. First, the cross-sectional case–control design precludes the establishment of causal relationships between urinary PPD-Q concentrations and colorectal cancer risk. Second, urinary PPD-Q concentration was measured using a single spot urine sample. Because urinary PPD-Q levels may vary over time with changes in environmental exposure, metabolism, and urinary excretion, a single measurement may not reliably represent cumulative exposure. This within-person temporal variability may have resulted in PPD-Q exposure misclassification. Repeated urine measurements would be required to characterize long-term PPD-Q exposure more accurately. Third, the study population was restricted to residents of a single city in China, which may affect the broader applicability of our findings to other geographic regions with different PPD-Q exposure profiles, lifestyles, and genetic backgrounds. Fourth, although the models were adjusted for several demographic and lifestyle characteristics, detailed data on important CRC-related factors, including physical activity, dietary fiber intake, red and processed meat consumption, and overall dietary patterns, were unavailable in this study. In addition, substantial differences in smoking and drinking habits were observed between CRC cases and controls. Although these variables were included in the multivariable models, they were assessed as categorical variables and may not fully capture the cumulative effects of tobacco and alcohol exposure, such as duration, intensity, and lifetime consumption. Therefore, residual confounding from smoking, drinking, and correlated lifestyle factors cannot be completely excluded. Fifth, the relatively modest sample size, especially for subgroup analyses, may have reduced statistical power to detect associations for certain PPD-Q congeners with lower detection frequencies or narrower exposure ranges. Lastly, while we employed BKMR to model mixture effects, the lack of toxicokinetic data for individual PPD-Qs may have influenced the estimation of their independent contributions. Future prospective cohort studies with repeated exposure measurements, larger and more diverse populations, and integrated toxicological investigations are warranted.

## 5. Conclusions

In this case–control study, higher urinary concentrations of 6PPD-Q, CPPD-Q, and DPPD-Q were associated with higher odds of CRC after adjustment for the measured covariates. BKMR analysis also suggested a positive joint association between the urinary PPD-Q mixture and CRC risk. These findings provide preliminary epidemiological evidence of an association between urinary PPD-Q biomarkers and CRC.

## Figures and Tables

**Table 1:** Demographic Features of Colorectal Cancer Cases and Controls.

	*n* (%) or Mean ± SD			
	Cases (*n* = 283)		Controls (*n* = 515)	
**Gender**				
Male	149	52.7%	266	51.7%
Female	134	47.3%	249	48.3%
**Age (years)**	60 ± 6.4		59 ± 9.7	
**Body mass index (BMI; kg/m^2^)**				
<18.5	39	13.8%	97	18.8%
18.5–25.5	185	65.4%	327	63.5%
>25.5	59	20.8%	91	17.7%
**Level of education**				
Below high school	88	31.1%	185	35.9%
High school	116	41.0%	224	43.5%
College	79	27.9%	106	20.6%
**Occupational status**				
Unemployed	75	26.5%	149	28.9%
Employed	208	73.5%	366	71.1%
**Marital status**				
Unmarried	46	16.3%	63	12.2%
Married	237	83.7%	452	87.8%
**Fast-food intake**				
<3 times/week	185	65.4%	403	78.3%
≥3 times/week	98	34.6%	112	21.7%
**Spicy food consumption**				
Occasional consumption	105	37.1%	206	40.0%
Regular consumption	178	62.9%	309	60.0%
**Residence**				
Urban region	228	80.6%	406	78.8%
Rural region	55	19.4%	109	21.2%
**Smoking habit**				
Nonsmoker	179	63.3%	458	88.9%
Smoker	104	36.7%	57	11.1%
**Drinking habit**				
Nondrinker	182	64.3%	447	86.8%
Drinker	101	35.7%	68	13.2%
**Family history of colorectal cancer**				
No	249	88.0%	486	94.4%
Yes	34	12.0%	29	5.6%

**Table 2:** Concentrations (μg/g Creatinine) of PPD-Qs in Urine from Colorectal Cancer Cases and Healthy Controls.

	6PPD-Q	77PD-Q	DPPD-Q	IPPD-Q	DTPD-Q	CPPD-Q
***Healthy controls***						
**DF**	79.3%	79.1%	73.7%	88.2%	78.0%	82.9%
**Mean**	1.37	0.68	0.58	0.70	0.40	0.37
**25th**	0.31	0.20	0.14	0.41	0.12	0.09
**50th**	0.68	0.74	0.31	0.58	0.23	0.20
**75th**	2.95	1.80	0.90	1.49	0.83	0.91
**Range**	<LOD–8.30	<LOD–2.68	<LOD–2.92	<LOD–4.06	<LOD–3.42	<LOD–2.72
***Colorectal cancer cases***						
**DF**	82.4%	80.3%	70.2%	89.6%	79.8%	80.7%
**Mean**	1.75	0.56	0.83	0.73	0.47	0.56
**25th**	0.28	0.17	0.31	0.39	0.15	0.11
**50th**	0.72	0.29	0.85	0.79	0.26	0.33
**75th**	2.79	0.96	1.21	2.46	1.44	0.83
**Range**	<LOD–13.3	<LOD–4.74	<LOD–3.58	<LOD–7.16	<LOD–7.07	<LOD–2.22

**Table 3:** Association Analysis of Urinary PPD-Q Concentrations with the Odds of Colorectal Cancer.

		Crude	Adjusted
		OR (95% CI)	OR (95% CI)
**6PPD-Q**	**Q1**	reference	reference
	**Q2**	1.17 (0.62, 1.72)	1.06 (0.56, 1.56)
	**Q3**	1.40 (1.02, 1.78)	1.53 (1.12, 1.94)
	**Q4**	1.64 (1.21, 2.07)	1.79 (1.26, 2.32)
	***p*** ** for trend**	<0.01	<0.01
**77PD-Q**	**Q1**	reference	reference
	**Q2**	0.64 (0.29, 0.99)	0.66 (0.31, 1.02)
	**Q3**	0.92 (0.60, 1.25)	1.01 (0.74, 1.28)
	**Q4**	0.62 (0.44, 0.81)	0.61 (0.37, 0.85)
	***p*** ** for trend**	0.37	0.28
**CPPD-Q**	**Q1**	reference	reference
	**Q2**	0.96 (0.58, 1.34)	1.03 (0.63, 1.43)
	**Q3**	1.30 (0.85, 1.75)	1.41 (1.07, 1.75)
	**Q4**	1.52 (1.12, 1.92)	1.60 (1.26, 1.94)
	***p*** ** for trend**	0.03	0.01
**DPPD-Q**	**Q1**	reference	reference
	**Q2**	1.05 (0.77, 1.34)	1.36 (0.85, 1.87)
	**Q3**	1.30 (1.07, 1.53)	1.22 (1.01, 1.43)
	**Q4**	1.51 (1.26, 1.76)	1.47 (1.25, 1.68)
	***p*** ** for trend**	0.02	0.03
**DTPD-Q**	**Q1**	reference	reference
	**Q2**	0.96 (0.63, 1.29)	0.96 (0.49, 1.43)
	**Q3**	1.27 (0.76, 1.78)	1.31 (0.78, 1.84)
	**Q4**	0.75 (0.26, 1.24)	0.68 (0.24, 1.12)
	***p*** ** for trend**	0.28	0.21
**IPPD-Q**	**Q1**	reference	reference
	**Q2**	1.02 (0.59, 1.45)	1.10 (0.64, 1.56)
	**Q3**	0.94 (0.31, 1.57)	0.91 (0.59, 1.23)
	**Q4**	0.84 (0.56, 1.12)	1.02 (0.32, 1.73)
	***p*** ** for trend**	0.51	0.49

## Data Availability

The original contributions presented in this study are included in the article/Supplementary Materials. Further inquiries can be directed to the corresponding author.

## Supplementary Materials

The following supporting information can be downloaded at https://www.mdpi.com/article/10.3390/toxics14080705/s1: Table S1: Abbreviation, Full Name, and CAS Number of Target PPD-Qs; Table S2: MRM Transition Parameters Used for Detecting PPD-Qs. Dwell Time was set at 0.05 s; Table S3: LODs and Extraction Recoveries of Target PPD-Qs in Human Urine; Table S4: Intra-day and Inter-day Relative Standard Deviations of PPD-Qs in Human Urine.

## Abbreviations

The following abbreviations are used in this manuscript:

PPD-Q	*p*-Phenylenediamine-quinone
QA/QC	Quality assurance/quality control
