# Supplementary material for: Associations Between Human Exposure to Substituted p-Phenylenediamine-Derived Quinones and Colorectal Cancer Risk
Source: Toxics. 2026 Aug 10;14(8):705. doi: 10.3390/toxics14080705 (PMC13517603; doi:10.3390/toxics14080705)
Supplement: Supplementary file 1 [file toxics-14-00705-s001.zip › toxics-4435348-supplementary.pdf]

Supporting Information

Associations between Human Exposure to Substituted *p*-Phenylenediamine-Derived Quinones and Colorectal Cancer Risk

Zhenling Fu[1], Zefu Hu[1], Hangbiao Jin[2], Sihui Yin*,[1]

[1.] Department of Pharmacy, Quzhou People's Hospital, The Quzhou Affiliated Hospital, Wenzhou Medical University, Quzhou, Zhejiang 324000, P. R. China.

[2.] State Key Laboratory of Green Chemical Synthesis and Conversion, Zhejiang Key Laboratory of Clean Energy Conversion and Utilization, Science and Education Integration College of Energy and Carbon Neutralization, Zhejiang University of Technology, Hangzhou, Zhejiang, 310014, P. R. China.

Table S1. Abbreviation, Full Name, and CAS Number of Target PPD-Qs.

Abbreviation	Full name	CAS	Supplier
**6PPD-Q**	2-((4-Methylpentan-2-yl)amino)-5-(phenylamino)cyclohexa-2,5-diene-1,4-dione	2754428-18-5	LGC Standards
**CPPD-Q**	2-(Cyclohexylamino)-5-(phenylamino)cyclohexa-2,5-diene-1,4-dione	68054-78-4	LGC Standards
**DPPD-Q**	2,5-Bis(phenylamino)cyclohexa-2,5-diene-1,4-dione	3421-08-7	AK Scientific
**IPPD-Q**	2-(Isopropylamino)-5-(phenylamino)cyclohexa-2,5-diene-1,4-dione	68054-73-9	LGC Standards
**DTPD-Q**	2,5-Bis(o-tolylamino)cyclohexa-2,5-diene-1,4-dione	252950-56-4	Cayman Chemical
**77PD-Q**	2,5-Bis((5-methylhexan-2-yl)amino)cyclohexa-2,5-diene-1,4-dione	Not available	J&K Scientific
**6PPD-Q-d_5**		Not available	LGC Standards
**Benzophenone-d_{10}**		Not available	J&K Scientific

Table S2. MRM Transition Parameters Used for Detecting PPD-Qs. Dwell Time was set at 0.05 Second.

	Quantitative transition (*m/z*)	Cone voltage (eV)	Collision energy (eV)	Qualitative transition (*m/z*)	Cone voltage (eV)	Collision energy (eV)
**6PPD-Q**	299/187	45	20	299/241	45	11
**CPPD-Q**	297/215	45	13	297/187	55	20
**DPPD-Q**	291/263	40	17	291/144	45	29
**IPPD-Q**	257/187	45	21	257/215	40	12
**DTPD-Q**	319/212	45	24	319/184	40	25
**77PD-Q**	335/237	50	20	335/167	55	25
**6PPD-Q-d_5**	304/191	45	25			
**Benzophenone-d_{10}**	193/82	40	22			

Table S3. LODs and Extraction Recoveries of Target PPD-Qs in Human Urine.

	LOD (ng/mL)	Extraction recovery (%) Spiked at 0.50 ng/mL		Spiked at 2.0 ng/mL		Spiked at 15 ng/mL	
		Mean	SD	Mean	SD	Mean	SD
**6PPD-Q**	0.051	90	5	88	3	81	8
**CPPD-Q**	0.039	81	9	92	10	105	11
**DPPD-Q**	0.020	104	4	108	6	96	6
**IPPD-Q**	0.088	94	3	93	8	99	5
**DTPD-Q**	0.016	95	7	106	4	92	11
**77PD-Q**	0.027	83	5	84	3	90	7

Table S4. Intra-day and Inter-day Relative Standard Deviations of PPD-Qs in Human Urine.

	Spiking at 0.5 ng/mL		Spiking at 5.0 ng/mL	
	**Intra-day variability**	**Inter-day variability**	**Intra-day variability**	**Inter-day variability**
**6PPD-Q**	3.6 ± 1.5	6.1 ± 2.1	6.3 ± 1.3	5.2 ± 2.1
**CPPD-Q**	7.5 ± 0.8	6.9 ± 3.8	7.9 ± 2.9	12 ± 4.0
**DPPD-Q**	8.1 ± 2.6	3.2 ± 1.6	12 ± 1.4	13 ± 1.8
**IPPD-Q**	3.0 ± 1.1	4.0 ± 2.7	8.4 ± 3.5	8.2 ± 4.3
**DTPD-Q**	8.7 ± 2.4	5.5 ± 2.0	8.3 ± 0.6	11 ± 3.3
**77PD-Q**	8.2 ± 1.3	8.1 ± 0.9	3.5 ± 2.6	7.5 ± 2.7
